# Molecular Advances on Cannabinoid and Endocannabinoid Research

**DOI:** 10.3390/ijms241612760

**Published:** 2023-08-14

**Authors:** Rosaria Meccariello

**Affiliations:** Department of Movement and Wellness Sciences, Parthenope University of Naples, 80133 Naples, Italy; rosaria.meccariello@uniparthenope.it

Since ancient times, cannabis has been used for recreational and medical purposes [1]. Nevertheless, it was only in the 1960s that Raphael Mechoulam and Yechiel Gaoni reported the structure of Δ^9^-tetrahydrocannabinol (Δ^9^-THC), the main psychotropic phytocannabinoid in the marijuana plant, *Cannabis sativa* L., [2]. This finding opened new gates for studies on the pharmacological and therapeutic potential of plant-derived cannabinoids, also known as phytocannabinoids. Currently, more than 550 chemical compounds have been reported in the marijuana plant, and more than 100 are phytocannabinoids with different properties and psychotropic or non-psychotropic effects (e.g., Δ^9^-THC and its isomer Δ^8^-THC, cannabinol (CBN) from THC degradation, cannabidiol (CBD), cannabichromene (CBC), cannabigerol (CBG), cannabinodiol (CBND), cannabigerolic acid (CBGA) and cannabielsoin (CBE)) [3]. 

Studies on Δ^9^-THC have been instrumental to the discovery of cannabinoid receptors and endogenous cannabinoids (i.e., endocannabinoids), revealing the existence of an evolutionarily conserved endogenous signaling system that is widely expressed in biological systems and that modulates many biological functions and tissue homeostasis. The endocannabinoid system (ECS) comprises the following elements: (i) endocannabinoids (e.g., *N*-arachidonoylethanolamide (AEA, also known as anandamide), 2-arachidonoylglycerol (2-AG), 2-arachidonylglyceryl ether (noladin ether, 2-AGE), virodhamine, etc.) and endocannabinoid-like substances (i.e., *N*-acylethanolamines and monoacylglycerols like oleoylethanolamide (OEA), palmitoylethanolamide (PEA), linoleoylethanolamide (LEA), or 2-oleoylglycerol); (ii) canonical and non-canonical cannabinoid receptors (e.g., type 1 cannabinoid receptor (CB1), type 2 cannabinoid receptor (CB2), GPR55, GPR119, transient receptor potential vanilloid type 1 (TRPV1), nuclear peroxisome proliferator-activated receptor γ (PPARγ)); biosynthetic and hydrolyzing enzymes (e.g., *N*-acylphosphatidylethanolamine-specific phospholipase D (NAPE-PLD) and diacylglycerol lipase (DAGL) for the biosynthesis of AEA and 2-AG, respectively; fatty acid amide hydrolase (FAAH) and monoacylglycerol lipase (MAGL)/FAAH for the hydrolysis of AEA and 2-AG, respectively); (iii) transporters (e.g., AEA intracellular carriers belonging to the fatty acid binding protein (FABP) family, or endocannabinoid membrane transporter (EMT)) [4]. The ECS has a recognized role in the control of synaptic plasticity, learning and memory, neurogenesis, neuroinflammation, pain control, stress response, mood and behavior, energy homeostasis, food intake and metabolism, reproduction, fertility and pregnancy, immune response, cardiac functions, cancer progression, etc. [5,6,7,8,9,10,11,12,13,14,15]. Furthermore, the development of agonists/antagonists/inhibitors of cannabinoid receptors, metabolic enzymes and transporters in parallel to the isolation of several psychotropic and non-psychotropic plant-derived cannabinoids has drawn attention to the development of cannabinoid-based therapies and their possible use in personalized medicine. Currently, recreational/medical use of cannabinoids is gaining traction and legitimacy in many countries, but the large distribution of cannabinoid receptors in biological systems and the pleiotropic activity of the ECS require further investigations.

This Special Issue of IJMS collected a total of eight manuscripts, seven research articles and one review article that collectively provide insights in the molecular advances in cannabinoid and endocannobinoid research.

The production of (phyto)cannabinoids from sources other than *C. sativa* is an intriguing question, with implications in terms of knowledge and bio-production. By means of a generated sequence similarity network (SSN), Go and coworkers [16] discovered three enzymes in *Brassica rapa* subsp. *pekinensis*, *Nicotiana attenuata* and *Papaver somniferum*, respectively, all belonging to the berberine bridge enzyme family, that can catalyze the oxidative cyclization of the monoterpene moiety in CBGA to form CBE. This finding not only fills a knowledge gap in the biosynthetic pathways of (phyto)cannabinoids, but also suggests an attractive methodological approach to discover new actors in the “cannabinoids world”, potentially applicable for their controlled bio-production. 

Preclinical data from animal models point out the potential efficacy of cannabis and cannabinoids for the management of cancer-related symptoms and of palliative and non-palliative pain in cancer patients. However, the real utility of cannabinoids, cannabis, or cannabis-based medicines in the treatment of cancer has yet to be convincingly demonstrated [17,18]. Despite this, studies on cell lines, animal models or biopsies have revealed the deregulation on the endocannabinoid system in cancer and that endocannabinoid signaling modulates multiple aspects of tumor development like cell growth, cell cycle progression, apoptosis of tumor cells, migration, invasion, metastasis, and tumor angiogenesis [13,14,19]. Currently, the pathophysiological implications of CB2 in cancer remain controversial. In their research article, Iden et al. [20] used selected animal models to investigate the hypothesis that CB2 might have a protective role in suppressing tumorigenesis in colorectal cancer. The authors demonstrate that CB2 signaling can modulate the immune response and reduce tumorigenesis, in turn. In parallel, the authors also examine the possible association of single nucleotide polymorphisms (SNPs) in *CNR2*, the gene encoding for the CB2 protein, with the incidence of colon cancer in the human population, revealing a significant association between the incidence of colon cancer and non-synonymous variants of *CNR2*. These findings further corroborate the anti-tumorigenic role of CB2 in colon cancer and suggest that this receptor might represent a promising target in cancer treatment. 

CB2 has been proposed as an anti-inflammatory target in several inflammatory and immune diseases [7]. In this respect, the pathogenesis of Duchenne Muscular Dystrophy (DMD), an X-linked dystrophinopathy due to a mutation in the *DMS* gene encoding for dystrophin, is characterized by muscular degeneration in conjunction with several secondary co-morbidities, such as cardiomyopathy, respiratory failure, and chronic inflammatory states [21]. Argenziano et al. [22] analyzed the effect of the CB2 agonist JWH-133 on DMD-associated primary macrophages, revealing that the pharmacological activation of CB2 counteracts inflammation by inhibiting the release of pro-inflammatory cytokines and by directing the phenotype of macrophages toward anti-inflammatory and immunosuppressive activities (i.e., M2 status). Hence, the authors highlight the promising possibility to target CB2 in DMD as an additional and effective anti-inflammatory therapy.

The research article by Aretxabala and coworkers [23] expands our knowledge of ECS activity in the nucleus, analyzing the subcellular distribution of DAGL-α in neurons, the enzyme that synthesizes 2-AG, namely the most abundant endogenous agonist of CB1. The authors demonstrate that 2-AG is produced in the nuclear matrix of nuclei derived from the adult rat cortex. In parallel, to find potential candidates in the regulation of 2-AG levels and signaling in the nuclear matrix, they analyzed the subcellular distribution of 2-AG metabolizing enzymes (i.e., MAGL, FAAH, α/β-hydrolase domain 12 protein (ABHD12) and cyclooxygenase-2 (COX2)). Interestingly, the authors suggest that ABHD12 is probably the most important serine hydrolase that affects 2-AG production in the nuclear matrix, thus including this enzyme in the list of the possible ECS targets with pathophysiological relevance.

Neuro-inflammation is critical in both brain aging and the pathogenesis of neurological diseases [24,25]. Learning deficit, reduced neurogenesis and enhanced pro-inflammatory glial activity are the pro-aging consequences of ECS impairment in the brain [11,12]. In this respect, Palmisano et al. [26] used a mouse model of site-specific deletion of the CB1 encoding gene, *CNR1*, within hippocampal neurons to investigate if the hippocampal aging, decreased neurogenesis and increased neuroinflammation observed in *CB1*^−/−^ knockout mice was an indirect consequence of generalized aging or the direct effect of CB1 depletion in hippocampal neurons. Their research article provides evidence that CB1 signaling locally affects brain aging, modulating both neurogenesis and the communication between neurons and glia. Hence, the direct activity of CB1 within the hippocampus maintains neurogenesis and prevents both neuroinflammation and age-related cognitive decline. 

In Western countries, the recreational use of illicit drugs, such as cannabis, is common mainly in male adolescents and young men, with several negative life outcomes like depression, substance use disorders, and psychosis, as well as social impacts [27,28]. In this respect, adolescence is a critical period for the completion of several developmental processes, including neurodevelopment and reproductive functions [15,28,29]. Hence, cannabis use during adolescence might epigenetically impact brain health, and thus deserves attention. In their research article, Qvist et al. [30] used a proteomic approach to study the consequences of mid-adolescent exposure to the synthetic cannabinoid WIN 55,212-2 in a male rat model, followed by drug abstinence in late adolescence. Attention has been focused on the changes in the protein levels in the prefrontal cortex, the brain region undergoing age-related thickness changes [31]. Out of a total of 487 deregulated proteins mainly related to the γ-aminobutyric acid (GABA)-ergic neurotransmitter system, the synaptic Ras GTPase-activating protein 1 (SYNGAP1) significantly increased, but also exhibited a specific spatio-temporal and developmental profile (e.g., SYNGAP1 increases only in synaptosomal fractions, but only after prolonged drug abstinence, and in adolescents). Interestingly, SYNGAP1 is a known biomarker of neurodevelopmental disorders [32], and the authors demonstrate that the protein is also increased in the prefrontal cortex of Δ^9^-THC exposed rats, thus suggesting that SYNGAP1 may be the link between adolescence cannabis use and adverse neuropsychiatric outcomes.

The last research article of this Special Issue by Heiliczer and coworkers [33] reports the specific profile of endocannabinoids (i.e., 2-AG and AEA, their endogenous breakdown product arachidonic acid, and the endocannabinoid-like compounds OEA and PEA) in the saliva of patients suffering different types of chronic orofacial pain. Correlation analyses between diagnosis, pain characteristics, and salivary endocannabinoids revealed that specific endocannabinoids in saliva may represent a specific signature for certain types of chronic orofacial pain (i.e., migraine, tension-type headache, trigeminal neuralgia, and burning mouth syndrome) to be used in pain management and to achieve more accurate diagnoses.

Opioids and cannabinoids are distinct drug classes that have been historically used in medicine, in combination or separately, as analgesic for the treatment of severe pain [34]. Currently, their reciprocal interaction in antinociception is relevant in personalized medicine for pain management. Nevertheless, both cannabinoid and opioid use and abuse can lead to drug addiction in that both endogenous cannabinoid and opioid systems are involved in the control of the dopaminergic circuitry responsible for both natural rewards and the rewarding aspects of nearly all drugs of abuse [35,36]. Hence, the need for an alternative intervention to prevent drug addiction has been reported. In this respect, the review article by Psarianos and coworkers [37] analyzes the beneficial effects of physical exercise in the treatment of patients with opioid use disorders (OUDs), suggesting that physical exercise generates a sequence of events, primarily including the modulation of endocannabinoid and opioid systems within the brain, which day-by-day leads to a gradual disengagement from addiction. As a consequence, physical exercise may represent a non-pharmacological intervention that sequentially modulates reward, inhibition, and stress circuits, preserves brain health and a patient’s overall quality of life, reduces anxiety, depression and the desire for drug use, thus leading to a gradual disengagement from addiction. 

Taken together, this Special Issue of IJMS provides new insights into the involvement of cannabinoids and endocannabinoids in the maintenance of health status and the onset of disease, suggesting new actors in the cannabinoid world, and providing new molecular mechanisms, diagnostic markers and targets in cancer, drug abuse and addiction, aging or pain management. 
